# Comparative study of the etiology of nosocomial bacteremic pneumonia in ventilated and non-ventilated patients: a 10-year experience in an institution

**DOI:** 10.1128/spectrum.01517-23

**Published:** 2023-09-12

**Authors:** Emilio Bouza, Helmuth Guillen-Zabala, Adriana Rojas, Gabriela Cañada, Emilia Cercenado, Carlos Sánchez-Carrillo, Cristina Díez, Luis Puente, Patricia Muñoz, Alicia Galar

**Affiliations:** 1 Department of Clinical Microbiology and Infectious Diseases, Hospital General Universitario Gregorio Marañón, Madrid, Spain; 2 Instituto de Investigación Sanitaria Gregorio Marañón, Madrid, Spain; 3 CIBERES (CB06/06/0058), CIBER Enfermedades Respiratorias, Madrid, Spain; 4 Department of Medicine, School of Medicine, Universidad Complutense de Madrid, Madrid, Spain; 5 Centro de Investigación Biomédica en Red de Enfermedades Infecciosas (CIBERINFEC), Madrid, Spain; 6 Department of Pneumology, Hospital General Universitario Gregorio Marañón, Madrid, Spain; Johns Hopkins Hospital, Baltimore, Maryland, USA

**Keywords:** pneumonia, bacteremia, multidrug-resistant microorganisms

## Abstract

The etiology of nosocomial pneumonia (NP) in non-mechanically ventilated patients [hospital-acquired pneumonia (HAP)] is poorly understood because of difficulties in obtaining reliable respiratory samples. While it is thought to resemble that of mechanically ventilated patients [ventilator-associated pneumonia (VAP)], few studies compare etiology in both groups. We compared the etiology of bacteremic NP (bNP) episodes in HAP and VAP over 10 years in our institution. We defined NP in adults according to the American Thoracic Society criteria. bNP episodes were defined as significant isolates in ≥1 blood culture concordant with ≥1 respiratory pathogen isolated with <7 days of difference. During 2010–2019, 188 patients were included. The comparison between HAP (*n* = 104) and VAP (*n* = 84) revealed the following factors: male sex (80.8%/63.1%; *P* < 0.01); median age (69.3/67.8 y-o; *P* = 0.602); etiology [*Staphylococcus aureus* (40.49%/21.4%; *P* < 0.01), Enterobacterales (35.6%/39.3%; *P* = 0.601), and *Pseudomonas aeruginosa* (14.4%/34.5%; *P* < 0.01)]. Microorganisms were considered multidrug-resistant in 29.8%/21.4% of cases, respectively (*P* = 0.193). Median hospital stay in HAP/VAP was 45.0/53.5 days (*P* = 0.255), mortality was 55.8%/53.6% (*P* = 0.770), and related mortality was 45.2%/35.7% (*P* = 0.233). The etiology of bNP in hospitalized patients is similar but not identical in HAP and VAP. The differences included a higher prevalence of *S. aureus* in HAP and *Pseudomonas aeruginosa* in VAP. bNP is a serious disease, with mortality >40%.

**IMPORTANCE:**

This study on bacteremic nosocomial pneumonia (bNP) demonstrates the importance of this condition both in patients undergoing and not undergoing mechanical ventilation. *Staphylococcus aureus*, Enterobacterales, and non-fermenting Gram-negative bacilli are all causative agents in ventilator-associated pneumonia (VAP) and hospital-acquired pneumonia (HAP), with a predominance of *S. aureus* in HAP and of *Pseudomonas aeruginosa* in VAP. Mortality in this condition is very high. Therefore, new therapeutic and preventive approaches should be sought.

## INTRODUCTION

The two classic types of hospital-acquired pneumonia are pneumonia acquired during mechanical ventilation [ventilator-associated pneumonia (VAP)] and pneumonia that develops in patients not undergoing mechanical ventilation [hospital-acquired pneumonia (HAP)] (1, 2).

The etiology of VAP is well known since, by definition, it occurs in intubated patients in whom lower respiratory tract samples are readily available for microbiological examination (3, 4). In contrast, data on the etiology of HAP are limited because the entity tends to be underreported, possibly because of challenges associated with specimen collection (1, 5). Furthermore, interpreting the significance of isolates in many sputum samples from HAP patients is difficult (6).

For the purposes of empirical treatment, it is often assumed that the etiologic agents in both groups of pneumonias are similar, although there is little evidence to support this assumption (1). Current empiric treatment guidelines for treatment of patients with HAP or VAP recommend vancomycin or linezolid for methicillin-resistant *Staphylococcus aureus* (MRSA) and piperacillin-tazobactam, an antipseudomonal cephalosporin, or a carbapenem for the treatment of *Pseudomonas aeruginosa*. In addition, these empiric regimens should consider the local distribution of pathogens associated with episodes and their antimicrobial susceptibilities (1).

The etiology of pneumonia is best confirmed when the same microorganism is isolated in blood and simultaneous lower respiratory tract samples, even if this situation is uncommon (1).

In our study, we compare episodes of bacteremic nosocomial pneumonia (bNP) in VAP and HAP patients in a general hospital over a 10-year period. We analyze the incidence of both entities, their etiologic agents, the presence of multidrug-resistant (MDR) microorganisms as causative pathogens, and the respective prognosis of both groups of patients.

## MATERIALS AND METHODS

### Study design, participants, and setting

This was a retrospective study of adult patients (>16 years) who were admitted to a tertiary-care hospital from January 2010 to December 2019.

We selected patients with bacteremia and clinical evidence of nosocomial pneumonia (NP), following the criteria of the American Thoracic Society (1). The patients had to have one or more significant positive blood cultures and at least one respiratory sample with the isolation of the same microorganism within 0–7 days of the positive blood culture. Patients were classified as HAP or VAP according to standard criteria (1), and only a single culture per admission was included for analysis.

Processed respiratory samples included sputum, tracheal aspirate, bronchial aspirate, and bronchoalveolar lavage fluid.

Sputum quality was determined by the proportion of leukocytes and epithelial cells. Sputum was considered acceptable with >25 leukocytes and <15 epithelial cells or with >15 leukocytes and <5 epithelial cells, both at low amplification. No such restrictions were applied in patients with severe neutropenia. The predominant potentially pathogenic bacteria were reported. The remaining types of respiratory samples were plated with a 2.5-µL calibrated loop, and bacteria present at more than 10^4^ CFU./mL were reported.

Patient data included age, sex, date of admission and discharge, underlying diseases, Charlson comorbidity index, McCabe and Jackson score, immunodepression status, symptoms and chest X-ray images during the episode, severity of the disease, bacterial isolates (identification and susceptibility), antimicrobial treatment, and clinical outcome.

Patients were considered to have severe diseases if they required one or more of the following: oxygen supplementation, mechanical ventilation, or admission to intensive care units due to the progress of the pneumonia. Respiratory insufficiency was defined as PaO_2_ less than 60 mm Hg or oxygen saturation less than 90% in room air. We considered empyema, meningitis, shock, and death during the first 30 days after hospital admission to be complications of bacteremic pneumonia.

We followed the CDC and ECDC standard terminology for MDR microorganisms that are important causes of HAP and VAP. MDR microorganisms were not susceptible to at least one agent in three or more antimicrobial categories (7). This group included *Pseudomonas* species resistant to carbapenems, quinolones, and third-generation cephalosporins, excluding combinations of cephalosporins with beta-lactamase inhibitors (i.e., ceftolozane-tazobactam and ceftazidime-avibactam). In addition, extended-spectrum beta-lactamase-producing Gram-negative bacilli and carbapenemase-producing Gram-negative bacilli were included. We also considered as MDR microorganisms all *Acinetobacter* species resistant to carbapenems or those resistant to at least three categories of antibiotics (piperacillin-tazobactam, third- and fourth-generation cephalosporins, quinolones, and aminoglycosides). All *Stenotrophomonas* and *Burkholderia* species were considered MDR owing to their intrinsic resistance to multiple antibiotic categories.

Difficult-to-treat microorganisms comprised those Gram-negative bacilli that were not susceptible to all agents in all beta-lactam categories, including carbapenems, or to fluoroquinolones but that were susceptible to the combination of cephalosporins and beta-lactamase inhibitors (8, 9).

Methicillin-resistant *Staphylococcus aureus* was considered an MDR microorganism.

All-cause mortality during hospital admission was recorded. Deaths related to bacteremic pneumonia comprised those that occurred during acute infection while the patients were receiving antimicrobial treatment for the episode of bNP, provided that there was no evident alternative cause.

A quality score for the adequacy of the antimicrobial treatment received was also determined for patients who met the study inclusion criteria (from 0 points if inadequate to 2 points if adequate for each of the five variables). The variables taken into account in the elaboration of the score were route of administration, dose and duration, adequate selection of antimicrobial treatment, and de-escalation when possible. The minimum overall score would be 0 points and the maximum 10 points.

The Ethics Committee of Hospital General Universitario Gregorio Marañón approved this study (Code: MICRO.HGUGM.2021–023) and authorized its performance.

### Data analysis

The median and interquartile range (IQR) or mean and standard deviation (SD) were used in the descriptive statistics for continuous variables. Categorical variables were compared using the χ^2^ test with a Yates correction or Fisher’s exact test, as necessary. Continuous variables were compared using the *t* test or the Mann-Whitney test when a normal distribution of the data could not be assumed.

Statistical significance was set at *P* < 0.05. All statistical analyses were performed using SPSS Statistics 21, IBM, Chicago, IL, USA.

## RESULTS

From January 2010 to December 2019, our institution recorded a total of 493,764 admissions of adult patients. Of these, 188 presented microbiologically confirmed bNP and fulfilled our inclusion criteria. Of these, 104 episodes were HAP and 84 VAP. Overall, the incidence of microbiologically confirmed bNP per 100,000 admissions during the study period was 21.1 episodes of HAP and 17.0 episodes of VAP (*P* = 0.625). A single culture per admission and per patient was counted for analysis.

The demographic and clinical characteristics of both groups (HAP and VAP) are compared in Table 1. The most common underlying disease in HAP and VAP patients was cardiovascular disease [67/104 (64.4%) and 55/84 (65.5%), respectively], followed by solid organ neoplasm [36/104 (34.6%) and 20/84 (24.8%), respectively], diabetes mellitus [27/104 (26.0%) and 19/84 (22.6%), respectively], chronic obstructive pulmonary disease (COPD)/bronchitis [24/104 (23.1%) and 18/84 (21.4%), respectively], and chronic kidney disease [23/104 (22.1%) and 15/84 (17.9%), respectively].

**TABLE 1:** Demographic and clinical characteristics of HAP and VAP patients with nosocomial bacteremic pneumonia episodes

	HAP patients (*n* = 104)	VAP patients (*n* = 84)	*P*
**Incidence of nosocomial bacteremic pneumonia/100,000 admissions**	21.1	17.0	0.625
**Age, median (IQR**)	69.3 (58.0–76.0)	67.8 (53.6–76.2)	0.602
**Male sex, *n* (%**)	84/104 (80.8%)	53/84 (63.1%)	**<0.01**
**Underlying disease, *n* (%**)			
Cardiovascular disease	67/104 (64.4%)	55/84 (65.5%)	1.0
Solid organ neoplasm	36/104 (34.6%)	20/84 (23.8%)	0.113
Diabetes	27/104 (26.0%)	19/84 (22.6%)	0.614
COPD^*a*^ bronchitis	24/104 (23.1%)	18/84 (21.4%)	0.861
Chronic kidney disease	23/104 (22.1%)	15/84 (17.9%)	0.584
Transplant	10/104 (9.6%)	1/84 (1.2%)	**0.024**
Hepatic cirrhosis	8/104 (7.7%)	5/84 (6.0%)	0.776
Hematological neoplasm	8/104 (7.7%)	4/84 (4.8%)	0.552
HIV	4/104 (3.8%)	3/84 (3.6%)	1.0
Autoimmune disease	4/104 (3.8%)	3/84 (3.6%)	1.0
Hemodialysis	3/104 (2.9%)	1/84 (1.2%)	0.630
Immunosuppressants	11/104 (10.6%)	4/84 (4.8%)	0.181
Corticosteroids	7/104 (6.7%)	4/84 (4.8%)	0.757
**Charlson score, median (IQR**)	5.0 (3.0–6.0)	4.0 (2.0–6.0)	0.079
**McCabe score**			
Rapidly fatal, *n* (%)	3/104 (2.9%)	2/84 (2.4%)	1.0
Ultimately fatal, *n* (%)	33/104 (31.7%)	23/84 (27.4%)	0.527
Non-fatal, *n* (%)	69/104 (66.3%)	59/84 (70.2%)	0.638
**Radiology pattern, *n* (%**)			
Bilateral involvement	37/104 (35.6%)	27/84 (32.1%)	0.621
Multilobar involvement	29/104 (27.9%)	19/84 (22.6%)	0.410
Presence of cavitations	4/104 (3.8%)	2/84 (2.4%)	0.570
Interstitial pattern	11/104 (10.6%)	9/84 (10.7%)	0.976
Alveolar pattern	45/104 (43.3%)	48/84 (57.1%)	0.059
Pleural effusion/empyema	17/104 (16.3%)	13/84 (15.5%)	0.871
**Need for supplemental oxygen, *n* (%**)	91/104 (87.5%)	84/84 (100.0%)	**<0.01**
**Respiratory insufficiency, *n* (%**)	87/104 (83.7%)	74/84 (88.1%)	0.412
**Complications of pneumonia, *n* (%**)	49/104 (47.1%)	41/84 (48.8%)	0.884
**Median days of hospital stay (IQR**)	45.0 (24.0–73.0)	53.5 (30.2–96.7)	0.255
**Median days under mechanical ventilation (IQR**)	11.0 (5.0–31.5)	18.0 (7.0–40.0)	0.095
**Median days in the ICU (IQR**)	12.5 (1.0–32.5)	28.0 (9.0–44.0)	**<0.01**
**Mortality, *n* (%**)	58/114 (55.8%)	45/101 (53.6%)	0.770
**Related mortality, *n* (%**)	47/114 (45.2%)	30/101 (35.7%)	0.233

^*a*^ COPD: chronic obstructive pulmonary disease.

Many patients in the HAP and VAP groups had an alveolar pattern [45/104 (43.3%) and 48/84 (57.1%), respectively] and bilateral involvement [37/104 (35.6%) and 27/84 (32.1%)] on the chest X-ray, and most required oxygen supplementation [91/104 (87.5%) and 84/84 (100%), respectively].

The proportion of episodes caused by different pathogens is summarized in Table 2. *Staphylococcus aureus* was significantly more frequent in HAP than in VAP (40.4% vs 21.4%, respectively; *P* < 0.01), while *P. aeruginosa* was more frequent in VAP than in HAP, where it caused one-third of all episodes (34.5% vs 14.4%, respectively; *P* < 0.01). No significant differences were found between HAP and VAP, respectively, for microorganisms such as Enterobacterales (35.6% vs 39.3%, *P* = 0.601), *Acinetobacter* sp. (3.8% vs 2.4%, *P* = 0.570), *Stenotrophomonas maltophilia* (1.0% vs 2.4%, *P* = 0.440), and other microorganisms (4.8% vs 0.0%, *P* = 0.066).

**TABLE 2:** Microbiological characteristics of nosocomial bacteremic pneumonia episodes in HAP and VAP patients

	HAP patients (*n* = 104)	VAP patients (*n* = 84)	*P*
**Etiology of episodes, *n* (%**)			
*Staphylococcus aureus*	42/104 (40.4%)	18/84 (21.4%)	**<0.01**
*Methicillin-resistant *Staphylococcus aureus*	18/42 (42.9%)	8/18 (44.4%)	0.909
Enterobacterales	37/104 (35.6%)	33/84 (39.3%)	0.601
*Pseudomonas aeruginosa*	15/104 (14.4%)	29/84 (34.5%)	**<0.01**
*Acinetobacter baumannii*	4/104 (3.8%)	2/84 (2.4%)	0.570
*Stenotrophomonas maltophilia*	1/104 (1.0%)	2/84 (2.4%)	0.440
Other microorganisms	5/104 (4.8%)	0/84 (0.0%)	0.066
*Multidrug-resistant Gram-negative bacilli	13/57 (22.8%)	10/66 (15.2%)	0.278
*Difficult-to-treat Gram-negative bacilli	2/57 (3.5%)	2/66 (3.0)	0.881
**Multidrug-resistant microorganisms**	31/104 (29.8%)	18/84 (21.4%)	0.193
**Polymicrobial infection, *n* (%**)	6/104 (5.8%)	6/84 (7.1%)	0.702
**Positive respiratory samples, *n* (%**)			
Bronchial aspirate	61/104 (58.7%)	74/84 (88.1%)	<0.01
Sputum	26/104 (25.0%)	0%	<0.01
Other (bronchoalveolar lavage and pleural fluid)	17/104 (16.3%)	10/84 (11.9%)	0.388

The types of respiratory samples in which pneumonia was confirmed in HAP and VAP episodes were, respectively, bronchial aspirate (58.7% vs 88.1%, *P* < 0.01), sputum (25.0% vs 0%, *P* < 0.01), and other (16.3% vs 11.9%, *P* = 0.388). Microorganisms causing pneumonia were considered MDR in 29.8% of HAP episodes and 21.4% of VAP episodes (*P* = 0.193).

Among *S. aureus* isolates, MRSA accounted for 42.9% (18/42) in the HAP group and 44.4% (8/18) in the VAP group (*P* = 0.909) (Table 1). Regarding Gram-negative microorganisms, 22.8% in the HAP group (13/57) and 15.2% in the VAP group (10/66) were MDR (*P* = 0.278). The most common MDR microorganisms were *Acinetobacter baumannii* (*n* = 6; four in HAP episodes and two in VAP episodes; *P* = 0.560), *P. aeruginosa* (*n* = 5; two in HAP episodes and three in VAP episodes; *P* = 0.400), and *Klebsiella pneumoniae* (*n* = 5; four in HAP episodes and one in VAP episodes; *P* = 0.231). We found four difficult-to-treat Gram-negative bacilli: three difficult-to-treat *P. aeruginosa* isolates (*n* = 3; one in HAP episodes and two in VAP episodes; *P* = 0.248) and one difficult-to-treat *Burkholderia cepacia* in a HAP patient.

Only six episodes involved polymicrobial bNP in HAP patients (6/104; 5.8%) and nine in VAP patients (9/84; 7.1%) (*P* = 0.702). Most patients started antimicrobial treatment in less than 1 hour for HAP episodes (77.9%) and VAP episodes (88.1%), with less than a 1-day delay until initiation of appropriate treatment (Table 3). The median duration of therapy was 14 days for both groups. The overall quality score regarding the adequacy of the antimicrobial treatment administered was 7.1 points out of 10 for HAP patients and 6.8 out of 10 for VAP patients (*P* = 0.958).

**TABLE 3:** Comparison of antimicrobial treatment for nosocomial bacteremic pneumonia episodes in HAP and VAP patients

Antimicrobial treatment	HAP patients (*n* = 104)	VAP patients (*n* = 84)	*P*
**Time to start treatment, *n* (%**)			0.371
<1 hour	81 (77.9)	74 (88.1)	
1–4 hours	7 (6.7)	3 (3.6)	
4–8 hours	2 (1.9)	0 (0)	
>8 hours	5 (4.8)	2 (2.4)	
Not available	9 (8.7)	5 (6.0)	
**Days of delay until correct treatment, median (IQR**)	0 (0–2)	0 (0–1)	0.145
**Treatment duration, median days (IQR**)	14 (8.5–18)	14 (9.7–16.2)	0.349
**Quality score of the treatment 0–2 (0: inadequate 2: adequate), mean (±SD**)			
Route of administration	1.98 (0.21)	1.89 (0.46)	0.183
Dose	1.93 (0.37)	1.72 (0.68)	0.011
Duration	0.95 (0.97)	1.03 (0.92)	0.514
Adequate antibiotic	1.62 (0.77)	1.54 (0.79)	0.443
De-escalation	0.63 (0.92)	0.67 (0.92)	0.653
*Overall score* (0–10)	7.11 (2.13)	6.85 (2.65)	0.958

Median hospital stay in patients with microbiologically confirmed bacteremic HAP and VAP was 45.0 vs 53.5 days (*P* = 0.255), respectively. Mortality in HAP and VAP groups was 55.8% vs 53.6% (*P* = 0.770), and mortality considered related to the episode was 45.2% vs 35.7% (*P* = 0.233), respectively.

## DISCUSSION

This study on bNP demonstrates the importance of this entity, both in patients who are undergoing mechanical ventilation and in those who are not. *S. aureus*, Enterobacterales, and non-fermenting Gram-negative bacilli share causality in both VAP and HAP, with a predominance of *S. aureus* in the HAP patients and *P. aeruginosa* in the VAP patients. Mortality in this entity is very high, and new therapeutic and preventive approaches should be sought.

The incidence density of VAP is well known in different circumstances, with figures ranging from 9 to 24 episodes per 1,000 days of mechanical ventilation (4, 10, 11). A recent decrease in the incidence of VAP attributable to different interventions has been suggested (12, 13), although changes in definition and surveillance methods make this trend doubtful (14 – 16).

The incidence of HAP is much less known. A North American multicenter study estimated 4.15–4.54 HAP episodes per 10,000 days of hospital stay and found no evidence of increases in recent years (17). In other studies, the denominator was hospital admissions rather than stays, and the incidence of HAP ranged from 1.6 to 18.8 cases per 1,000 admissions (18 – 21).

Our incidence data are not comparable with previous data since we only included microbiologically proven cases with the simultaneous presence of bacteremia. Our figures of 0.21 and 0.17 cases of bacteremic VAP and HAP per 1,000 admissions, respectively, represent only the subgroup of fully microbiologically confirmed cases.

Data on the frequency of bacteremia in patients with VAP are scarce in the literature and have been estimated in some studies at between 12% and 18% (22 – 24). On the other hand, in patients with HAP, we were unable to find global figures on the frequency of bacteremic episodes outside specific studies of various microorganisms. For example, in patients with staphylococcal pneumonia, bacteremia was recorded in 47.5% of patients with COVID-19 and in only 6.3% of those with other underlying diseases (25).

The main objective of our study, however, was to compare etiology between patients with bacteremic VAP and HAP within the same institution and over a long period of time. We found that the microorganisms were the same in both groups but with quantitative differences in favor of *S. aureus* and *P. aeruginosa* in HAP and VAP, respectively.

In line with data reported elsewhere, our study confirms that the main causative microorganisms in VAP are *S. aureus*, Enterobacterales, and non-fermenting Gram-negative bacilli (NFGNB) (mainly *P. aeruginosa*) (3, 4, 26 – 29). In the case of HAP, data on etiology have traditionally been scarce or non-existent owing to difficulties in obtaining reliable lower respiratory samples in most patients (1, 17, 18). This is the reason why we included patients whose etiology was confirmed by blood and respiratory cultures. Our data suggest that the etiologic agents are very similar to those of VAP and clearly different from those reported in a series of community-acquired pneumonia (30 – 32). The high relevance of *S. aureus* in HAP in our series and the lower proportion of *P. aeruginosa* infections than in VAP are remarkable.

As expected, MDR is a serious problem for both Gram-positive and Gram-negative bacteria causing bNP. In the case of bNP due to *S. aureus*, the isolates in some series were MRSA in more than 40% of episodes, as we found in our study (33, 34). In the case of bNP caused by NFGNB, the proportion of MDR strains was also high, particularly when the causative agents are *P. aeruginosa* and *A. baumannii* (35). We found that the percentage of MDR among bNP caused by Gram-negative bacilli reached 18%, with *P. aeruginosa* and *A. baumannii* accounting for 9% and that the percentage of difficult-to-treat microorganisms was low.

Lack of coverage for MDR microorganisms in the choice of empirical treatment can lead to a worse clinical outcome (1). In this sense, the recommendations of the current guidelines suggest a choice of empirical treatment based on clinical data and the antimicrobial resistance patterns of the isolates at the institution. However, bacteremic HAP and VAP are not unusual (22, 34, 36, 37). Therefore, the definitive identification of a pathogen, often MDR, in blood culture might alter management and provide further guidance for both antimicrobial treatment and treatment de-escalation in HAP and VAP. We believe that blood cultures and respiratory samples should be obtained in all episodes of nosocomial pneumonia—both HAP and VAP—in order to improve antimicrobial treatment.

The mortality of nosocomial pneumonia is high. However, in bacteremic patients, such as those assessed here, it exceeded 40%, with no significant differences between VAP and HAP. Bacteremia in patients with bNP seems to increase the risk of death (22, 28, 29, 33, 34, 38 – 41). In this context, the need to prolong the duration of antimicrobial treatment in bacteremic patients is an unresolved issue (42 – 44) .

Our study is limited by the fact that it represents the experience of a single institution and by its retrospective nature, although it is not biased by any other form of case selection.

We attempted to draw attention to the etiology of HAP in a general hospital over a long period of time. The microorganisms causing HAP can be deduced from those causing VAP. They are very often pathogens that are difficult to treat because of their MDR nature. The findings of our study support the need to perform blood cultures in all hospitalized patients with NP and to use anti-MRSA and anti-MDR NFGNB antibiotics as empirical treatment for nosocomial pneumonia in HAP and VAP patients.

## AFTER EPUB

*[This article was published on 12 September 2023 with Pablo Martín-Rabadán included in the byline. The byline was updated in the current version, posted on 3 November 2023.]*
